# Acute Deep Vein Thrombosis in Venous Aneurysm following Closure of the Chronic Traumatic Arteriovenous Fistulae of the Lower Extremities

**DOI:** 10.1155/2016/1375214

**Published:** 2016-05-18

**Authors:** Saranat Orrapin, Supapong Arworn, Kittipan Rerkasem

**Affiliations:** ^1^Division of Vascular and Endovascular Surgery, Department of Surgery, Faculty of Medicine, Chiang Mai University, Chiang Mai 50200, Thailand; ^2^Research Institute of Health Science, Chiang Mai University, Chiang Mai University, P.O. Box 80, Chiang Mai 50202, Thailand

## Abstract

Chronic traumatic arteriovenous fistula (AVF) commonly results from an unrecognized vascular injury. In this report, there were two cases of chronic traumatic AVF of the legs with a long history of stab (case 1) and shotgun wounds (case 2). Both cases presented with varicose veins together with hyperpigmentation around the ankle of the affected leg. Angiograms showed a single large AVF in case 1, whereas, in case 2, there was a single large AVF together with multiple small AVFs. In both cases large venous aneurysm was found next to a large AVF. An open surgical AVF closure for the large AVF was performed in case 1 successfully, but patient developed acute deep vein thrombosis (DVT) in a large venous aneurysm. In the second case, in order to prevent DVT, only closure of the large AVF was performed, which preserved arterial flow into the venous aneurysm. Case 2 did not have acute DVT. This report raised the concern about acute DVTs in venous aneurysms following the closure of chronic traumatic AVF in terms of prevention. Also chronic traumatic AVF is commonly due to misdiagnosis in the initial treatment, so complete and serial physical examinations in penetrating vascular injury patients are of paramount importance.

## 1. Introduction

Chronic traumatic arteriovenous fistula (chronic traumatic AVF) is a condition that commonly results from an unrecognized or untreated minor vascular trauma. In the past this condition occurred most commonly in military situations, often relating to penetrating gunshot wounds, but, at the present time, due to the wide use of firearms, the condition is found frequently in civilian cases [1]. Around 60% of untreated or unrecognized vascular injuries may develop into chronic traumatic AVF [2]. Robbs et al. reported that 63% of chronic traumatic AVF cases were due to stab wounds, 26% due to shotgun wounds, and only 1% due to blunt trauma [3].

AVF affects various organs in the body, especially the cardiovascular system. It depends on the size and site of AVF and the cardiovascular condition of the patient [4]. If AVF was unrecognized or left untreated, it often progressed and developed the signs and symptoms of chronic AVF, such as proximal venous or arterial aneurysm, limb swelling and hypertrophy, chronic venous disease, vascular steal syndrome, or high output cardiac failure [5, 6].

The principle treatment was to close the AVF and restore normal vascular flow. However, the chronic changes of an AVF may affect large proximal venous arms of AVF (venous aneurysm), so abrupt interruption of fast flow from AVF into such large veins caused acute postoperative deep vein thrombosis (DVT) [7]. There are still no standard guidelines of prevention or management. This report shows 2 cases with lower extremity chronic traumatic AVF patients at Chiang Mai University Hospital and raised the concern about acute DVT following AVF closure. This study was approved by our local ethic committee.

## 2. Clinical Presentation

### 2.1. Case  1

A 61-year-old Thai male arrived at the hospital presenting with a right lower abdominal pulsatile expansile mass. He also had a varicose vein on his right leg and hyperpigmented skin on his right ankle. He did not have any underlying conditions, such as connective tissue disorder, aneurysmal related genetic disorder, or thrombophilic disease.

He had history, 20 years ago, of a stab wound in his right thigh with massive bleeding. He received treatment by suturing skin at a hospital. Two years later he noticed that his right leg had swollen and the skin around right ankle was hyperpigmented. During the last 5 years he felt a pulsatile mass developing in the right side of his lower abdomen, but he had no pain on this mass.

A physical examination revealed a pulsatile and expansile mass, 15 cm in diameter, in the right side of his lower abdomen, but he had no pain. His right leg showed signs of chronic venous insufficiency (CVI), namely, a varicose vein, lipodermatosclerosis, and a swollen leg. There was thrill and a pulse in his varicose vein.

An abdominal ultrasound and duplex scan of the right leg were carried out. There was a pulsatile, high flow wave in the IVC, right iliac vein, and right femoral vein with a suspected AVF in the thigh area due to a previous injury.

**Figure 1:**
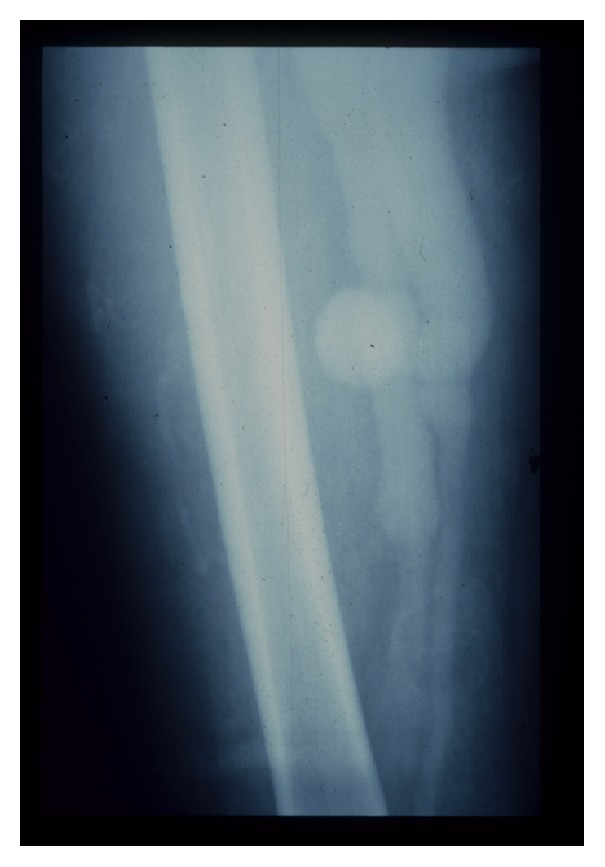
Angiogram showed the arteriovenous fistula at mid-superficial femoral artery and vein with large venous aneurysm next to fistula.

**Figure 2:**
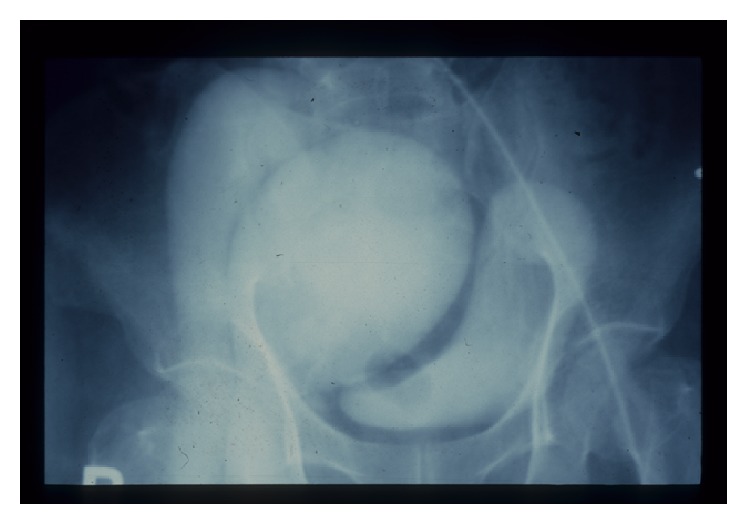
Angiogram presented the large aneurysm of common iliac artery and vein.

Then an angiogram was performed. A large thigh chronic AVF with proximal artery and venous aneurysm were detected at midthigh (Figure 1). A large venous aneurysm of superficial femoral vein (SFV) (2.5 cm in diameter) together with pseudoaneurysm of superficial femoral artery (SFA) next to the fistula was also found. This angiogram showed a common iliac artery aneurysm and a common iliac vein aneurysm (18 cm in diameter) (Figure 2).

The patient was operated on to correct his symptoms by resection of AVF together with an arterial segment. The SFA was repaired by end-to-end anastomosis of unaffected segment and the SFV was repaired by suturing the venous defect. Soon after the operation, patient suffered from acute DVT in the venous aneurysm of the SFV, which necessitated a postoperative anticoagulant and a graduated stocking for 2 years. Patient's CVI symptoms improved but still needed close surveillance for DVT.

### 2.2. Case  2

A 60-year-old male presented with chronic right side heart failure which had started around 2 years before. He had been diagnosed with chronic traumatic AVF at the right thigh due to a 30-year-old shotgun wound. Initially, CVI of the right leg had slowly developed. Two years before he had symptoms of right side heart failure, including leg swelling and liver congestion. These symptoms persisted and progressed into severe congestive heart failure (CHF). His echocardiography showed severe tricuspid regurgitation, mild mitral regurgitation, mild pulmonary regurgitation, and pulmonary hypertension.

**Figure 3:**
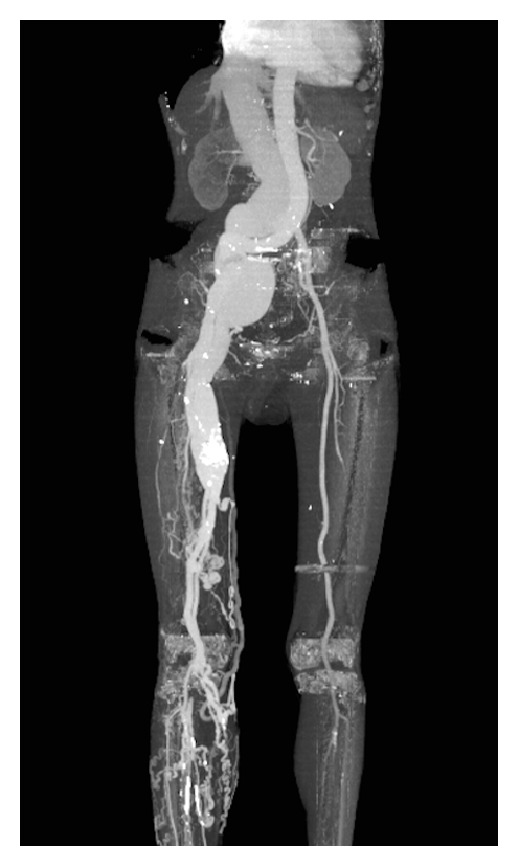
Computed tomographic angiogram of the patient shows an aneurysmal dilatation of the artery and vein proximal to the largest AVF in the midthigh. There were also multiple AVFs along the right leg from the external iliac artery to the distal superficial femoral artery.

A physical examination showed swelling of his right leg with hyperpigmented skin around the ankle. Pulsatile varicose veins in the right thigh and groin were observed. A CT angiogram showed one large AVF between mid-SFA and SFV together with multiple AVFs along the external iliac artery and vein, the common femoral artery and femoral vein, and the distal SFA and SFV. There was a 5 cm aneurysmal change in the proximal right external iliac artery and vein. A venous aneurysm (3 cm in diameter) was found next to the largest AVF site (Figure 3).

**Figure 4:**
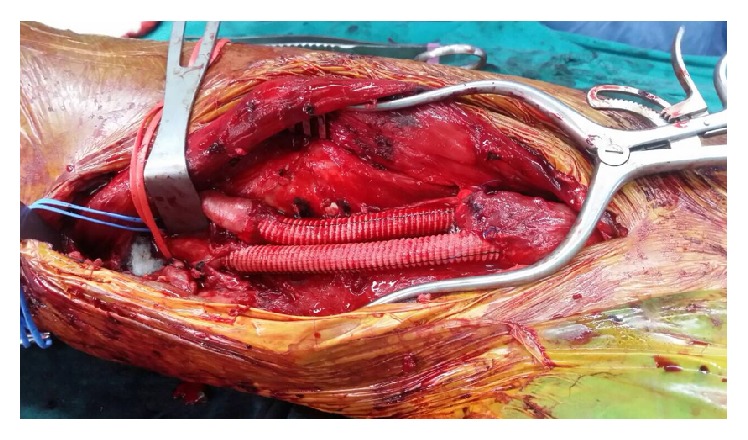
Intraoperative findings of the 2nd case. Chronic traumatic AVF and part of superficial femoral artery and femoral vein was resected. Interposition Dacron graft was placed.

At first, the authors planned to close all AVFs in this patient, but it was impossible because of multiple AVFs along the external iliac artery to the distal SFA level. Since the authors had an experience of postoperative DVT in the venous aneurysm in case 1 after closure of the chronic AVF, the authors closed only the largest AVF, which would facilitate a decrease in his cardiac return. This would ease his cardiac failure condition but still allow some arterial blood flow to prevent acute thrombosis in the venous aneurysm. This was carried out by resecting the dilated AVF segment, and then arterial and venous bypasses were performed using an 8 mm Dacron graft individually (Figure 4). Immediately after the authors clamped the AVF, the patient suffered reflex bradycardia (the Branham effect). The patient's heart rate dropped below 40 beat per min and his blood pressure decreased to 80/40 mmHg, so the anesthesiologist used a subcutaneous pacemaker to control his heart rate to 60 bpm, thereby increasing his blood pressure to around 100/60 mmHg.

After the operation his right side heart failure condition improved. His leg was less swollen and his cardiac pumping function improved. He was weaned off the percutaneous cardiac pacemaker within 1 week of the operation. He had no DVT or thrombosis of either the external iliac artery aneurysm or the external iliac vein aneurysm. He was able to return to work within 3 weeks of the operation but still needed surveillance ultrasound for venous aneurysm progression.

## 3. Discussion

A chronic traumatic AVF is a condition which is related to unrecognized or untreated penetrating vascular injury. This condition may lead to many physiological changes in the patient, ranging from local effects, such as varicose veins, to serious systemic effects, such as congestive heart failure. Treatment of chronic traumatic AVF might be difficult because of local changes in the artery and vein from proximal aneurysm dilatation, chronic changes of the AVF wall site, blood pooling around the operative site, or the patient's systemic condition, such as right side heart failure. Even now endovascular aneurysm repair is becoming more and more popular in the aorta. One might hypothesize that insertion of cover stent graft over fistula could manage the pathology. This is not so easy, because of the huge difference in diameter between the proximal and distal arteries to the fistula that is commonly found.

Closing all AVFs seemed like the best treatment option, but there was a chance of postoperative DVT complications due to sluggish blood flow in venous aneurysms, which can lead to massive pulmonary embolisms due to large thrombus in such aneurysm. Patients therefore would need to have postoperative DVT prophylaxis. Firstly, on the ground, in case of multiple traumatic AVF (shotgun wounds), the authors performed an alternative operation, closing only the large AVF and leaving small AVFs (case 2). This corrected the patient's symptoms and may have prevented postoperative DVT, perhaps lowering the chance of a future venous aneurysm rupture. Such patients need long term surveillance. This hypothesis is based on a venous bypass, such as Palma's operation, utilizing the femorofemoral venous bypass, which was carried out to correct the unilateral iliac vein occlusion. This operation adds a small AVF in the groin at the inflow site to avoid stasis of the blood in the graft [8]. Also our team reported a case recently where the patient had an aortocaval fistula for 40 years and was treated using endovascular therapy [9]. This patient had a large venous aneurysm in IVC (20 cm in diameter). After endovascular graft landing, a small endoleak developed, but, in a way, this created a fast flow through the huge venous aneurysm, which avoided DVT. Secondly, pneumatic compression and elevation of patients' leg should be in place in early postoperative period. Lastly authors recommended heparinizing patients by using either unfractionated heparin or low molecular weight heparin (Enoxaparin) in the early postoperative period and changed to warfarin later. In case the patients did not have any indication for further warfarin usage, this medication could be stopped when patients were fully mobile such as 3 months after operation. We did not have any experience in inserting caval filter for pulmonary embolism prophylaxis in our series. This is because the IVC diameter was very large (>10 cm) in both case, so the chance of migration was expected to be high.

In chronic traumatic cases, complications such as severe reflex bradycardia (the Branham effect) from abrupt increase in peripheral vascular resistance should be anticipated and readily managed after AVF closure. Therefore, pacemakers must be readily available in operating theaters. Also operators should test whether patients might have such problems by temporarily clamping the fistula to see whether any cardiovascular disturbance happens.

To avoid these chronic traumatic AVF problems with misdiagnosis, early recognition by complete and serial physical examinations in penetrating vascular injury patients needs to be ensured. If any AVFs are detected, immediate repairs should be carried out in order to reduce the risk of systemic complications.

## 4. Conclusion

Complete and serial physical examinations in penetrating vascular injury patients are important in reducing the incidence of chronic traumatic AVF in patients. If chronic traumatic AVF presents, AVF repair is recommended as soon as possible in order to reduce the complications from both AVF and the operation itself. DVT prophylaxis especially needs to be in place. In cases of multiple AVFs together with large venous aneurysms, as a good policy to prevent acute DVT in venous aneurysm, the authors propose closure of only the major fistula.
